# A pathology-based surrogate model for chemotherapy decision-making in intermediate-risk luminal breast cancer: validation of histologic grade and Ki67 in a Chinese population

**DOI:** 10.3389/fmed.2026.1727768

**Published:** 2026-02-05

**Authors:** Guanhong Li, Yi Zeng, Yu Sun

**Affiliations:** Department of Breast Surgery, The Affiliated Huizhou Hospital, Guangzhou Medical University, Huizhou, Guangdong, China

**Keywords:** adjuvant chemotherapy, breast cancer, Chinese population, estrogen receptor, progesterone receptor

## Abstract

**Introduction:**

Breast cancer requires precise decisions regarding postoperative adjuvant chemotherapy, as these choices critically influence both survival rates and quality of life. Although genetic testing has enhanced risk stratification, its limited accessibility in grassroots healthcare settings stems from high costs and ongoing debates regarding Western-derived models’ suitability for Chinese populations. This is particularly evident in intermediate-risk luminal-subtype patients without lymph node metastasis, where therapeutic controversies persist. Existing prediction models based on routine immunohistochemical markers face limitations due to data bias from Western cohorts, insufficient standardization of parameters, and interference from heterogeneous high-risk factors. Our study aims to validate clinicopathological predictors of chemotherapy responsiveness (including histological grade and a Ki67 index ≥20%), with the ultimate goal of developing a cost-effective predictive model specifically designed for Chinese patients.

**Methods:**

This retrospective study included surgically treated intermediate-risk breast cancer patients from Huizhou Third Municipal Hospital (2015–2022). It compared survival outcomes between chemotherapy-treated and chemotherapy-naïve cohorts. Propensity score matching (PSM) was applied to balance baseline disparities. The Kaplan–Meier method and Cox regression analyses were employed to assess disease-free survival (DFS) and overall survival (OS).

**Results:**

This study enrolled 476 intermediate-risk breast cancer patients (chemotherapy group: 288 cases; non-chemotherapy group: 186 cases), with a median follow-up of 57.5 months. After propensity score matching (196 cases), multivariate analysis identified histological grade 2–3 (HR = 5.398, *p* = 0.028), a Ki67 index ≥20% (HR = 5.020, *p* = 0.012), and absence of chemotherapy (HR = 3.998, *p* = 0.014) as independent risk factors for DFS. Chemotherapy significantly reduced the risk of recurrence in the Ki67 ≥ 20% subgroup (6.5% vs. 34.9%, *p* = 0.003) and the histological grade 2–3 subgroup (7.1% vs. 35.6%, *p* = 0.007), but it showed no significant benefit for the patients with a Ki67 index <20% or grade 1 (*p* > 0.05). In OS analysis, only a Ki67 index ≥20% emerged as an independent prognostic factor (HR = 9.301, *p* = 0.035), with no statistically significant difference observed for chemotherapy (*p* = 0.085).

**Conclusion:**

Our findings validate the histological grade and a Ki67 index ≥ 20% as predictive biomarkers of chemotherapy responsiveness in intermediate-risk luminal-subtype breast cancer. This evidence-based stratification offers clinical guidance for resource-constrained settings, while further prospective validation and integration with emerging biomarkers are warranted to refine precision therapeutic strategies.

## 1 Introduction

Breast cancer is the malignant tumor with the highest incidence among women worldwide, and decisions regarding postoperative adjuvant chemotherapy directly impact patients’ survival outcomes and quality of life (1).

The Chinese Anti-Cancer Association (CACA) guidelines classify postoperative breast cancer patients into low-risk, intermediate-risk, and high-risk categories. In early-stage luminal breast cancer, low-risk patients can achieve a favorable prognosis with endocrine therapy alone, whereas high-risk patients require chemotherapy. However, the optimal treatment strategy for intermediate-risk patients (e.g., those with luminal subtype tumors, pT2N0 disease, or a Ki67 index ≥20%) remains controversial. Current international and Chinese guidelines recommend utilizing genetic testing, such as Oncotype DX, MammaPrint, and PAM50, to inform chemotherapy decision-making in intermediate-risk Luminal breast cancer (2). These assays enable precise stratification of recurrence risk, thereby helping to avoid both overtreatment and undertreatment (3).

When genetic testing guidance is unavailable, clinical decisions frequently depend on standard clinicopathological characteristics. Ki67 serves as a crucial marker for tumor cell proliferation; however, no universally accepted optimal cut-off value exists, with multiple proposals including 10, 14, and 20% (4). Research indicates that tumors exhibiting high Ki67 levels could have increased sensitivity to chemotherapy (5), which may lead to survival benefits (6). Additionally, a higher histological grade usually suggests poorly differentiated tumor cells and enhanced invasiveness (7). Despite research efforts to construct predictive models for chemotherapy responsiveness based on pathological characteristics, such as Ki67 and histological grade, to replace genetic testing and decrease the financial burden (8), the majority of these models were not designed to account for the distinct features of the Chinese population. Furthemore, the assessment and interpretation of critical biomarkers such as Ki67 are hindered by a lack of standardized protocols, which compromises their predictive specificity and accuracy in intermediate-risk cohorts (9).

Based on the aforementioned background, this study aims to address two key objectives: (1) to identify clinicopathological predictors of chemotherapy benefit in intermediate-risk, node-negative, luminal-type breast cancer patients in the absence of genetic testing; and (2) to explore predictive models based on routine immunohistochemical markers to guide chemotherapy decisions as an alternative to genetic testing.

## 2 Materials and methods

### 2.1 Study design and patients

This retrospective cohort study analyzed breast cancer patients treated at Huizhou Third People’s Hospital between January 2015 and January 2022. According to the CACA guidelines, patients were classified as low risk if all of the following criteria were met: pT ≤ 2 cm, histological grade 1, negative lymphovascular invasion (LVI), HER2 negativity, age >35 years, ER/PR positivity, and a Ki67 index <20%. High-risk status was defined by the presence of lymph node metastasis. Patients who did not meet the criteria for either low- or high-risk categories were classified as intermediate risk in accordance with guideline definitions (3). The inclusion criteria were as follows: (1) histologically confirmed invasive ductal carcinoma; (2) node-negative disease classified as stage IA–IIA according to the AJCC 8th edition; (3) hormone receptor-positive status (ER/PR ≥ 10%), and HER2-negative disease [including HER2-low (IHC 1 + or 2+/ISH−) and HER2-zero (IHC 0) tumors]; (4) age between 36 and 70 years; and (5) no prior ovarian function suppression (OFS) therapy. The exclusion criteria included the following: (1) prior 21−/70-gene panel testing, (2) receipt of neoadjuvant therapy or diagnosis of secondary malignancies, (3) non-surgical management, and (4) omission of adjuvant radiotherapy following breast-conserving surgery. Patients received four cycles of intravenous epirubicin (100 mg/m^2^) and cyclophosphamide (600 mg/m^2^) (EC regimen), administered every 3 weeks. Alternatively, patients received four cycles of intravenous docetaxel (75 mg/m^2^) and cyclophosphamide (600 mg/m^2^) (TC regimen), administered every 3 weeks. Premenopausal patients received tamoxifen (TAM), whereas postmenopausal patients received aromatase inhibitors (AIs). Throughout the treatment period, patients exhibited good compliance, and no treatment discontinuation occurred due to severe adverse events.

Clinicopathological data were extracted from electronic medical records and pathology databases, including age at diagnosis, menopausal status, surgical approach (with mandatory post-lumpectomy radiotherapy), AJCC pathological T/N staging, histological grade (Bloom-Richardson system), chemotherapy regimen, LVI, perineural invasion (PNI), hormone receptor expression levels, HER2 status (IHC/ISH results), Ki67, adjuvant endocrine therapy protocols, disease-free survival (DFS), and overall survival (OS). This study was conducted in accordance with the Declaration of Helsinki and the Good Clinical Practice guidelines, with ethical approval granted by the Institutional Review Board of Huizhou Third People’s Hospital.

All immunohistochemistry (IHC) slides were reviewed in a double-blinded manner by three specialized breast pathologists. In accordance with the 2018 American Society of Clinical Oncology/College of American Pathologists (ASCO/CAP) guidelines (10), the percentage of tumor cells stained for estrogen receptor (ER) and progesterone receptor (PR) expression was reported. Ki67 assessment was performed using the standardized “typewriter” visual assessment method, as recommended by the 2021 “International Working Group on Ki67 Assessment in Breast Cancer Guidelines” (11). According to the integrated 2024 Chinese Society of Clinical Oncology (CSCO) guidelines, IHC 0 was defined as no staining or ≤10% of invasive carcinoma cells showing incomplete, faint cell membrane staining; IHC 1 + was defined as >10% of invasive carcinoma cells showing incomplete, faint cell membrane staining; and IHC 2 + was defined as >10% of invasive carcinoma cells showing weak-to-moderate complete cell membrane staining or ≤10% of invasive carcinoma showing strong, complete cell membrane staining. Discordant cases were resolved through consensus review using a multiheaded microscope. Cases with an IHC score of 2 + (borderline) underwent reflex *in situ* hybridization (ISH) testing, all of which were confirmed as negative (12). To account for the potential impact of HER2 status on patient prognosis, cases were stratified into HER2-low and HER2-zero categories. The threshold for the key pathological indicator in this study strictly followed the current CACA guidelines, which designate Ki67 ≥ 20% as indicative of high tumor proliferation, and this threshold was adopted as the grouping standard (2).

### 2.2 Study endpoints and definitions

In this study, disease-free survival (DFS) served as the primary endpoint, while overall survival (OS) was the secondary endpoint. DFS was calculated from the date of definitive surgery to the earliest occurrence of: (1) histologically/radiologically confirmed locoregional recurrence or distant metastasis, (2) biopsy-proven contralateral primary breast cancer, or (3) all-cause mortality. OS was defined as the time from pathological diagnosis to all-cause mortality, with survivors censored at the date of last follow-up. Follow-up was primarily conducted via telephone inquiries, with information from hospital and clinic records. Follow-up ended on 30 November 2024. The choice of OS over breast cancer-specific survival (BCSS) was methodologically driven, given the inherent limitations of cause-of-death attribution in retrospective studies. OS, as an objective endpoint, minimizes attribution bias, according to STROBE Item 12, whereas BCSS necessitates prospective adjudication by oncology/cardiology review panels.

### 2.3 Statistical analysis

Statistical analyses were performed using SPSS (version 29.0; IBM Corp.) and the SangerBox platform (version 3.0) (10). Categorical variables were compared using Pearson’s chi-squared test. Propensity score matching (PSM) was performed using a 1:1 nearest neighbor algorithm to balance baseline characteristics, incorporating age, menopausal status, histological grade (Bloom-Richardson), pathological T stage, PR expression status, HER2 status, Ki67, and LVI as covariates. A caliper width of 0.03 was applied to ensure comparability. Survival probabilities were estimated using the Kaplan–Meier method, with between-group differences assessed using the log-rank test. Multivariable Cox proportional hazards regression models were constructed to evaluate the independent associations of variables with DFS and OS. A two-tailed *p*-value of <0.05 defined statistical significance throughout the analysis. The stability of the model was assessed using the bootstrap resampling method.

## 3 Results

The study cohort included 476 eligible patients, of whom 288 cases received adjuvant chemotherapy and 186 were managed with endocrine therapy alone. The median follow-up duration was 57.5 months (range 32–128 months). Table 1 summarizes the baseline demographic and clinicopathological characteristics of the chemotherapy and non-chemotherapy cohorts. Both cohorts demonstrated comparable distributions of surgical procedures (breast-conserving surgery vs. mastectomy), PNI, and endocrine therapy regimens, with corresponding *p*-values of 0.580, 0.122, and 0.711. The observed disparities in baseline characteristics reflected the established selection criteria for chemotherapy at our institution.

**Table 1:** Baseline characteristics of patients with chemotherapy and non-chemotherapy.

Characteristic	Adjuvant chemotherapy		*p* value
	YES *n* = 288	NO *n* = 186	
Age (years)			0.015
<50	166 (57.64%)	86 (46.24%)	
≥50	122 (42.36%)	100 (53.76%)	
Menstrual status			0.006
Premenopausal	180 (62.50%)	90 (48.39%)	
Postmenopausal	108 (37.50%)	96 (51.61%)	
Breast surgery			0.580
BCS	100 (34.72%)	60 (32.26%)	
Mastectomy	188 (65.28%)	126 (67.74%)	
T Stage			
1	114 (39.58%)	146 (78.49%)	<0.001
2	174 (60.42%)	40 (21.51%)	
Histologic grade			<0.001
1	108 (37.50%)	124 (66.67%)	
2–3	180 (62.50%)	62 (33.33%)	
PR			<0.001
≥20	220 (76.39%)	166 (89.25%)	
<20	68 (23.61%)	20 (10.75%)	
HER2 Status			<0.001
Low	212 (73.61%)	132 (70.97%)	
Zero	76 (26.39%)	54 (29.03%)	
Ki67			<0.001
<20%	104 (36.11%)	134 (72.04%)	
≥20	184 (63.89%)	52 (27.96%)	
Neural invasion			0.122
Yes	70 (24.31%)	34 (18.30%)	
No	218 (75.69%)	152 (81.70%)	
Vascular invasion			<0.001
Yes	116 (40.28%)	24 (12.90%)	
No	172 (59.72%)	162 (87.10%)	
Endocrine therapy			0.711
TAM	166 (57.64%)	104 (55.91%)	
AI	122 (42.36%)	82 (44.09%)	

HER2, human epidermal growth factor receptor 2; PR, progesterone receptor; BCS Breast conserving surgery; TAM, Tamoxifen; AI, Aromatase inhibitors.

A total of 40 DFS events and 18 OS events were documented. Univariate Cox regression analysis revealed that both DFS and OS were influenced by histological grade and Ki67. The surgical approach was significantly associated with OS only (Table 2). Variables with significant results in the univariate analysis were included in the multivariate Cox regression model. The results demonstrated that Ki67 was an independent risk factor for both DFS (HR = 3.813, 95% CI 1.568–9.275, *p* = 0.003) and OS (HR = 5.618, 95% CI 1.256–25.128, *p* = 0.024). High histological grade was an independent risk factor for DFS (HR = 4.572, 95% CI 1.586–13.178, *p* = 0.005), but it was not independently associated with OS (*p* = 0.153) (Table 3). Furthermore, compared to HER2-zero status, HER2-low status did not demonstrate independent prognostic value.

**Table 2:** Univariate Cox proportional hazard model of disease-free survival (DFS) and overall survival (OS) in all patients.

Factors	Disease-free survival		Overall survival	
	HR (95% CI)	*p*-value	HR (95% CI)	*p*-value
Age (years)				
<50	1 (Reference)		1 (Reference)	
≥50	1.040 (0.557, 1.939)	0.902	1.482 (0.556, 3.951)	0.431
Menstrual status				
Premenopausal	1 (Reference)		1 (Reference)	
Postmenopausal	1.504 (0.808, 2.797)	0.198	0.756 (0.298, 1.918)	0.556
Breast surgery				
BCS	1 (Reference)		1 (Reference)	
Mastectomy	0.582 (0.313, 1.082)	0.087	0.367 (0.142, 0.948)	0.038
T Stage				
1	1 (Reference)		1 (Reference)	
2	1.273 (0.684,2.368)	0.446	1.754 (0.690, 4.457)	0.238
Histologic grade				
1	1 (Reference)		1 (Reference)	
2–3	6.970 (2.477, 19.612)	<0.001	4.678 (1.073, 20.386)	0.040
PR				
≥20			1 (Reference)	
<20	1.299 (0.545, 3.095)	0.554	0.036 (0.001, 6.249)	0.207
HER2 Status				
Low	1 (Reference)		1 (Reference)	
Zero	1.403 (0.732, 2.688)	0.308	1.701 (0.666, 4.347)	0.267
Ki67				
<20%	1 (Reference)		1 (Reference)	
≥20	5.717 (2.399, 13.623)	<0.001	7.707 (1.771, 33.533)	0.006
Neural invasion				
Yes	1 (Reference)		1 (Reference)	
No	0.568 (0.289, 1.119)	0.102	0.443 (0.166, 1.181)	0.104
Vascular invasion				
Yes	1 (Reference)		1 (Reference)	
No	1.247 (0.660, 2.355)	0.496	0.692 (0.258, 1.857)	0.464
Endocrine therapy				
TAM	1 (Reference)		1 (Reference)	
AI	1.845 (0.989, 3.441)	0.054	1.290 (0.508, 3.274)	0.592
Chemotherapy				
Yes	1 (Reference)		1 (Reference)	
No	1.501 (0.800, 2.813)	0.206	0.900 (0.337, 2.405)	0.834

HER2, human epidermal growth factor receptor 2; PR, progesterone receptor; BCS Breast conserving surgery; TAM, Tamoxifen; AI, Aromatase inhibitors.

**Table 3:** Multivariate Cox proportional hazard model of disease-free survival (DFS) and overall survival (OS) in all patients.

Factors	Disease-free survival		Overall survival	
	HR (95% CI)	*p*-value	HR (95% CI)	*p*-value
Age (years)				
<50	ND		ND	
≥50	ND	ND	ND	ND
Menstrual status				
Premenopausal	ND		ND	
Postmenopausal	ND	ND	ND	ND
Breast surgery				
BCS	ND		1 (Reference)	
Mastectomy	ND	ND	0.398 (0.154, 1.030)	0.058
T Stage				
1	ND		ND	
2	ND	ND	ND	ND
Histologic grade				
1	1 (Reference)		1 (Reference)	
2–3	4.572 (1.586, 13.178)	0.005	2.977 (0.668, 13.274)	0.153
PR				
≥20	ND		ND	
<20	ND	ND	ND	ND
HER2 Status				
Low	ND		ND	
Zero	ND	ND	ND	ND
Ki-67				
<20%	1 (Reference)		1 (Reference)	
≥20	3.813 (1.568, 9.275)	0.003	5.618 (1.256, 25.128)	0.024
Neural invasion				
Yes	ND		ND	
No	ND	ND	ND	ND
Vascular invasion				
Yes	ND		ND	
No	ND	ND	ND	ND
Endocrine therapy				
TAM	ND		ND	
AI	ND	ND	ND	ND
Chemotherapy				
Yes	ND		ND	
No	ND	ND	ND	ND

HER2, human epidermal growth factor receptor 2; PR, progesterone receptor; BCS Breast conserving surgery; TAM, Tamoxifen; AI, Aromatase inhibitors.

To ensure comparability between the groups, 1:1 PSM with a caliper width of 0.03 was performed, resulting in a matched cohort of 196 patients. After propensity score matching, 21 DFS events and 11 OS events were recorded. The demographic and tumor characteristics between the two groups were balanced (Table 4). A total of 70 patients received TC chemotherapy, 28 received AC chemotherapy, and 98 did not receive chemotherapy. The methodology for PSM and the corresponding standardized mean differences (SMDs) are provided in Supplementary material 1. Subsequently, Cox regression analysis was performed on the matched dataset.

**Table 4:** Baseline characteristics of patients with chemotherapy and no-chemotherapy in PSM group.

Characteristic	Adjuvant chemotherapy		*p* value
	YES *n* = 98	NO *n* = 98	
Age (years)			0.465
<50	57 (58.16%)	62 (63.27%)	
≥50	41 (41.84%)	36 (36.73%)	
Menstrual status			0.463
Premenopausal	58 (59.18%)	63 (64.29%)	
Postmenopausal	40 (40.82%)	35 (35.71%)	
Breast surgery			>0.999
BCS	30 (30.61%)	29 (29.60%)	
Mastectomy	68 (69.39%)	69 (70.40%)	
T Stage			0.539
1	65 (66.33%)	80 (81.63%)	
2	33 (33.67%)	18 (18.37%)	
Histologic grade			0.666
1	56 (57.14%)	53 (54.08%)	
2–3	42 (42.86%)	45 (45.92%)	
PR			0.830
≥20	85 (86.73%)	86 (87.76%)	
<20	13 (13.27%)	12 (12.24%)	
HER2 Status			0.637
Low	71 (72.45%)	30 (30.61%)	
Zero	27 (27.55%)	68 (69.39%)	
Ki67			0.667
<20%	52 (53.06%)	55 (56.12%)	
≥20	46 (46.94%)	43 (43.88%)	
Neural invasion			0.123
Yes	26 (27.08%)	17 (17.35%)	
No	72 (72.92%)	81 (82.65%)	
Vascular invasion			0.611
Yes	21 (21.42%)	24 (24.49%)	
No	77 (78.58%)	74 (75.51%)	
Endocrine therapy			0.661
TAM	58 (59.18%)	61 (62.24%)	
AI	40 (40.82%)	37 (37.76%)	

HER2, human epidermal growth factor receptor 2; PR, progesterone receptor; BCS Breast conserving surgery; TAM, Tamoxifen; AI, Aromatase inhibitors.

Univariate analysis identified three significant predictors of DFS: higher histological grade (HR = 10.567, 95% CI: 2.460–45.384; *p* = 0.002), elevated Ki67 (≥20%; HR = 8.058, 95% CI: 2.364–27.474; *p* < 0.001), and chemotherapy omission (HR = 5.816, 95% CI: 1.952–17.329; *p* = 0.002). Multivariable Cox regression confirmed that these three factors were independent prognosticators: histological grade 2–3 (adjusted HR = 5.398, 95% CI: 1.202–24.252; *p* = 0.028), elevated Ki67 (≥20%; aHR = 5.020, 95% CI: 1.427–17.663; *p* = 0.012), and non-receipt of chemotherapy, which conferred a 3.998-fold increased risk of recurrence (95% CI: 1.322–12.088; *p* = 0.014). Internal validation using 500 bootstrap samples reported that all three factors showed statistical significance at a *p*-value of < 0.05, confirming the robustness of the results (Supplementary material 2A). Variables that did not reach significance in univariate analysis (*p* ≥ 0.05)—including age, menopausal status, surgical approach (breast-conserving surgery vs. mastectomy), T category, PR/HER2 expression levels, PNI, LVI, and endocrine therapy regimen (tamoxifen vs. aromatase inhibitors)—were not included in the multivariable model (Table 5).

**Table 5:** Cox proportional hazard model of disease-free survival (DFS) in PSM group.

Factors	Disease-free survival			
	Univariate analysis		Multivariate analysis	
	HR (95% CI)	*p*-value	HR (95% CI)	*p*-value
Age (years)				
<50	1 (Reference)		ND	ND
≥50	1.027 (0.425, 2.481)	0.954	ND	ND
Menstrual status				
Premenopausal	1 (Reference)		ND	ND
Postmenopausal	0.661 (0.280, 1.560)	0.345	ND	ND
Breast surgery				
BCS	1 (Reference)		ND	ND
Mastectomy	0.446 (0.189, 1.051)	0.065	ND	ND
T Stage				
1	1 (Reference)		ND	ND
2	0.589 (0.243, 1.430)	0.242	ND	ND
Histologic grade				
1	1 (Reference)		1 (Reference)	
2–3	10.567 (2.460, 45.384)	0.002	5.398 (1.202, 24.252)	0.028
PR				
≥20	1 (Reference)		ND	ND
<20	0.812 (0.189, 3.488)	0.779	ND	ND
HER2 Status				
Low	1 (Reference)		ND	ND
Zero	1.387 (0.572, 3.364)	0.469	ND	ND
Ki67				
<20%	1 (Reference)		1 (Reference)	
≥20	8.058 (2.364, 27.474)	<0.001	5.020 (1.427, 17.663)	0.012
Neural invasion				
Yes	1 (Reference)		ND	ND
No	0.585 (0.227, 1.509)	0.267	ND	ND
Vascular invasion				
Yes	1 (Reference)		ND	ND
No	0.633 (0.262, 1.530)	0.310	ND	ND
Endocrine therapy				
TAM	1 (Reference)		ND	ND
AI	1.506 (0.639, 3.550)	0.349	ND	ND
Chemotherapy				
Yes	1 (Reference)		1 (Reference)	
No	5.816 (1.952, 17.329)	0.002	3.998 (1.322, 12.088)	0.014

HER2, human epidermal growth factor receptor 2; PR, progesterone receptor; BCS Breast conserving surgery; TAM, Tamoxifen; AI, Aromatase inhibitors.

In contrast, OS was designated as an exploratory endpoint. During the entire follow-up period, only 11 events were recorded in the matched cohort. Univariate analysis of OS revealed significant prognostic value for both histological grade and the Ki67 proliferation index. The patients with histological grade 2–3 exhibited a 9.8-fold higher risk of mortality compared to those with grade 1 (HR = 9.800, 95%CI 1.254–76.589, *p* = 0.030). The patients with a Ki67 index ≥20% demonstrated a 12.8-fold higher risk of mortality compared to those with a Ki67 index <20% (HR = 12.819, 95%CI 1.638–100.356, *p* = 0.015). The no-chemotherapy group showed a marginal trend toward increased mortality risk (HR = 3.208, *p* = 0.085), although this did not reach statistical significance. Multivariate Cox regression revealed that high Ki67 expression was significantly associated with worse DFS (HR = 9.301, 95%CI:1.167–74.116, *p* = 0.035). Elevated histological grade demonstrated a clinically relevant, albeit non-significant, risk trend (HR = 6.474, *p* = 0.077). Internal validation using 500 bootstrap replications confirmed the maintained statistical significance of Ki67 (adjusted *p* = 0.016) and established a significant risk association for histological grade (adjusted *p* = 0.030). All predictors had 95% confidence intervals excluding unity, supporting the model’s robustness in DFS prediction (Supplementary material 2B). Of note, the limited sample size and the paucity of OS events resulted in extremely wide confidence intervals. Consequently, these findings should be considered exploratory and interpreted with caution (Table 6).

**Table 6:** Cox proportional hazard model of overall survival (OS) in PSM group.

Factors	Overall survival			
	Univariate analysis		Multivariate analysis	
	HR (95% CI)	*p*-value	HR (95% CI)	*p*-value
Age (years)				
<50	1 (Reference)		ND	ND
≥50	0.633 (0.168, 2.389)	0.500	ND	ND
Menstrual status				
Premenopausal	1 (Reference)		ND	ND
Postmenopausal	1.456 (0.443, 4.782)	0.536	ND	ND
Breast surgery				
BCS	1 (Reference)		ND	ND
Mastectomy	0.404 (0.123, 1.323)	0.134	ND	ND
T Stage				
1	1 (Reference)		ND	ND
2	2.334 (0.706, 7.722)	0.165	ND	ND
Histologic grade				
1	1 (Reference)		1 (Reference)	
2–3	9.800 (1.254, 76.589)	0.030	6.474 (0.819, 51.179)	0.077
PR				
≥20	1 (Reference)		ND	ND
<20	0.041 (0.000, 152.552)	0.447	ND	ND
HER2 Status				
Low	1 (Reference)		ND	ND
Zero	1.172 (0.339, 4.053)	0.802	ND	ND
Ki67				
<20%	1 (Reference)		1 (Reference)	
≥20	12.819 (1.638, 100.356)	0.015	9.301 (1.167, 74.116)	0.035
Neural invasion				
Yes	1 (Reference)		ND	ND
No	0.948 (0.205, 4.390)	0.945	ND	ND
Vascular invasion				
Yes	1 (Reference)		ND	ND
No	0.718 (0.209, 2.461)	0.598	ND	ND
Endocrine therapy				
TAM	1 (Reference)		ND	ND
AI	1.434 (0.437, 4.702)	0.552	ND	ND
Chemotherapy				
Yes	1 (Reference)		ND	ND
No	3.208 (0.850, 12.105)	0.085	ND	ND

HER2, human epidermal growth factor receptor 2; PR, progesterone receptor; BCS Breast conserving surgery; TAM, Tamoxifen; AI, Aromatase inhibitors.

**Figure 1:**
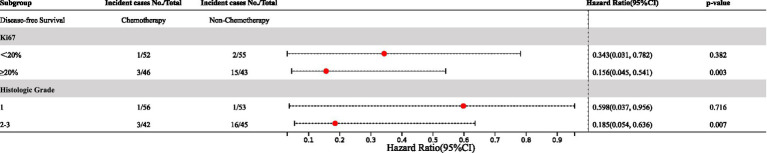
Cox proportional hazards model for disease-free survival (DFS) in the PSM subgroup.

Subgroup analysis based on Ki67 and histologic grade showed that, in the Ki67 ≥ 20% subgroup, the recurrence rate was 6.5% (3/46) in the chemotherapy group, significantly lower than 34.9% (15/43) in the non-chemotherapy group (HR = 0.156, 95% CI: 0.045–0.541, *p* = 0.003). In the histological grade 2–3 subgroup, the recurrence rate was 7.1% (3/42) in the chemotherapy group, significantly lower than 35.6% (16/45) in the non-chemotherapy group (HR = 0.185, 95% CI: 0.054–0.636, *p* = 0.007). Conversely, in the Ki67 < 20% subgroup (1.9% in the chemotherapy group vs. 3.6% in the non-chemotherapy group, *p* = 0.382) and the histological grade 1 subgroup (≤1.9% recurrence in both groups, *p* = 0.716), chemotherapy showed no statistical benefit (Figure 1).

**Figure 2:**
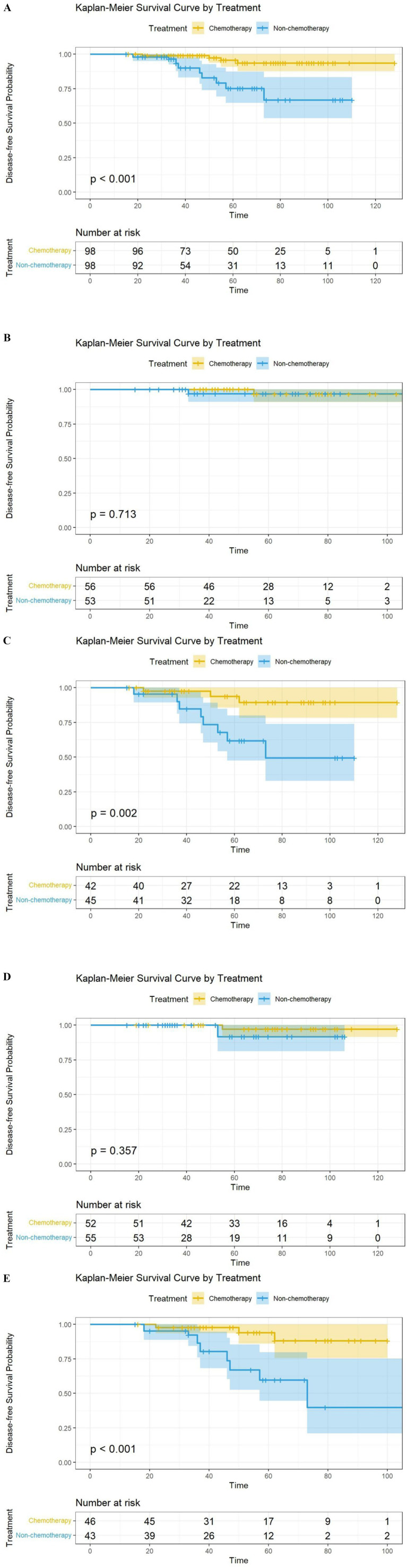
Kaplan–Meier curves and log-rank test results for disease-free survival in patients with **(A)** PSM group, **(B)** G1, **(C)** G2-G3, **(D)** Ki-67 < 20% and **(E)** Ki-67 ≥ 20%.

**Figure 3:**
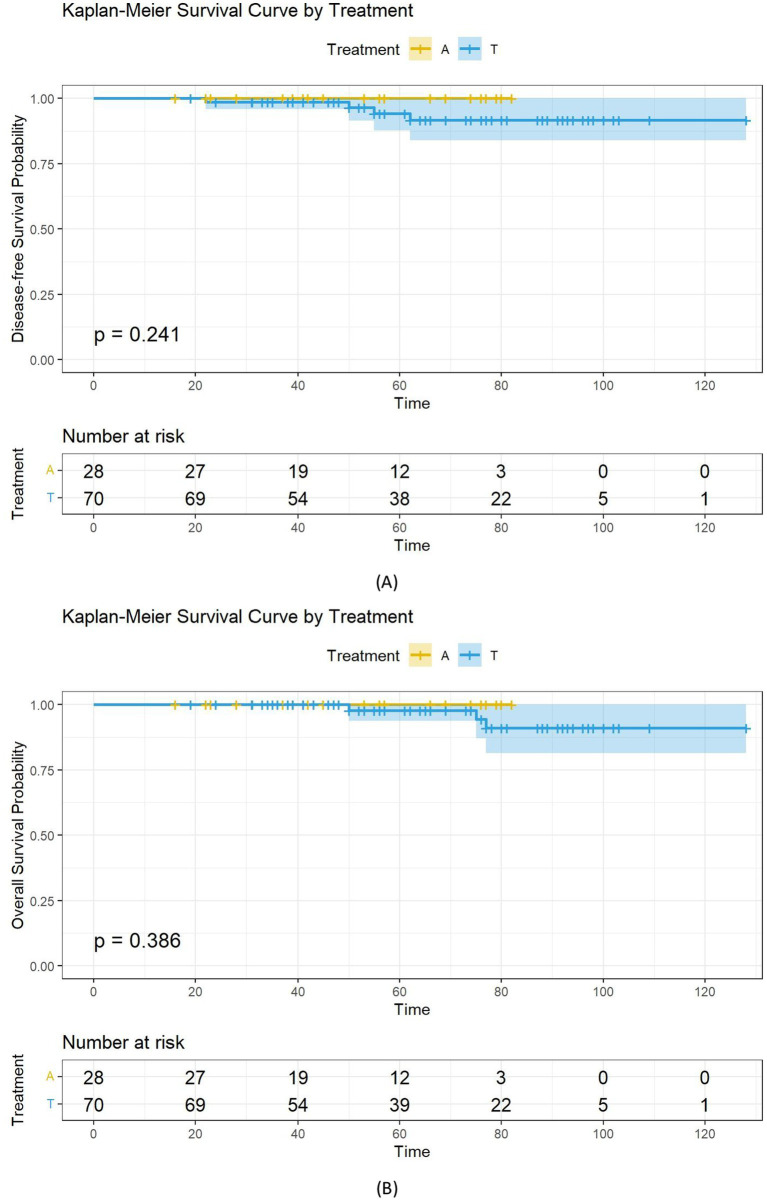
Kaplan–Meier survival curves for disease-free survival and overall survival, stratified by chemotherapy regimen. Kaplan-Meier curves and log-rank test findings of disease-free survival and overall survival in patients with **(A)** disease-free survival, **(B)** overall survival.

Kaplan–Meier analysis showed that the chemotherapy group had superior DFS (*p* < 0.001). Multivariate Cox regression analysis for the DFS endpoint found significant differences in Ki67 and histological grade. Based on these findings, we stratified the patients by Ki67 and histological grade in the matched cohort and performed Kaplan–Meier analysis for DFS. The Ki67 < 20% (*p* = 0.210) and G1 (*p* = 0.090) subgroups showed no chemotherapy benefit, while the patients in the chemotherapy group had improved DFS in the Ki67 ≥ 20% and G2-G3 subgroups (*p* < 0.001) (Figure 2). No significant difference in DFS or OS was observed between the AC and TC regimens within the chemotherapy group (all *p* > 0.05) (Figure 3).

In supplementary analyses, the impact of age and tumor size as continuous variables on DFS and OS was evaluated using Cox regression models. Age, analyzed as a continuous variable, demonstrated no statistically significant association with either DFS or OS. Tumor size was a significant independent predictor for both DFS and OS. In DFS analysis, univariate analysis showed that each 1 cm increase in tumor size significantly increased the risk of recurrence by 83.7% (HR = 1.837, 95% CI:1.248–2.705, *p* = 0.002). In multivariate models, after adjusting for other significant factors, this association remained significant, with a slightly higher HR (HR = 1.936, 95% CI:1.194–3.137, *p* = 0.007) (Supplementary material 3). Regarding OS, tumor size was also a significant prognostic factor: Univariate analysis yielded HR = 2.161 (95% CI:1.288–3.628, *p* = 0.004) and multivariate analysis yielded HR = 2.143 (95% CI:1.204–3.814, *p* = 0.010), indicating that each 1 cm increase more than doubled the risk of mortality (Supplementary material 4).

Furthermore, we systematically evaluated potential interactions between chemotherapy and endocrine therapy regimens by including interaction terms between these therapies in Cox regression models. Forest plots were used to visualize the interaction effects: For OS interaction, the HR was 0.92 (95%CI:0.75–1.14), and for DFS interaction, the HR was 1.03 (95%CI:0.82–1.30), with both 95%CIs crossing the null value (HR = 1). The absence of significant interaction terms indicates that the type of endocrine therapy (AI or TAM) did not modify the effect of chemotherapy on survival outcomes (Supplementary material 5).

## 4 Discussion

This study targeted a patient population defined by the CACA guidelines as having intermediate-risk, node-negative, luminal-type breast cancer. It systematically evaluated the predictive utility of standard clinicopathological characteristics for adjuvant chemotherapy benefit in settings where genetic testing is inaccessible. The pathology-based model developed in this study, incorporating histological grade and Ki67, demonstrated significant value in predicting DFS benefit for this intermediate-risk luminal breast cancer cohort. Subsequent stratification analysis revealed that chemotherapy significantly reduced recurrence risk in patients with high proliferative activity (HR = 0.156) or moderate/poor differentiation (HR = 0.185). Conversely, patients with low proliferation or well-differentiated tumors derived no significant benefit from chemotherapy. However, the limited number of OS events was insufficient to achieve the necessary statistical power for robust survival analysis. Consequently, the OS findings cannot support definitive conclusions and should be interpreted as exploratory.

At present, significant discrepancies persist among major guidelines regarding the optimal Ki67 cutoff value. The CACA guidelines adopt a Ki67 ≥ 20% threshold as a primary criterion for both disease subtyping and determining the need for chemotherapy (2). This approach, based on an inexpensive and readily available IHC marker, aligns effectively with the heterogeneous distribution of healthcare resources across China’s extensive territories. Conversely, owing to substantial inter-laboratory and inter-observer discrepancies in Ki67 evaluation and the absence of a unified detection protocol, the St. Gallen Consensus recommends a prudent approach. It suggests employing institution-specific medians or quartiles as thresholds and emphasizes the importance of integrated assessment incorporating histological grade (13). The NCCN guidelines, however, place greater emphasis on genetic testing. A high Ki67 index is regarded merely as a supplementary consideration for chemotherapy when genetic testing is inaccessible, with its contribution to final decision-making being subordinate to the results of genetic testing (14). Within this context, the present study was conducted in accordance with the CACA guidelines to evaluate the predictive performance of the Ki67 ≥ 20% cutoff value in a Chinese cohort of intermediate-risk luminal breast cancer patients. This study contributes to the development of diagnostic and therapeutic protocols that are better tailored to the Chinese context by utilizing pathological surrogate models, while adhering to the principle of precision medicine.

The results of this study strengthen the established biological association between tumor proliferation activity and sensitivity to chemotherapy. The intrinsic mechanism is driven by the fact that chemotherapeutic drugs principally target cells that are actively progressing through the cell cycle. As a biomarker directly correlating with the cellular proliferation fraction, a high Ki67 index implies that a greater proportion of tumor cells are in cell-cycle phases susceptible to the cytotoxic effects of chemotherapy (15, 16). Consequently, luminal B-like tumors characterized by high proliferation activity derive a more pronounced benefit from cytotoxic chemotherapy. This observation aligns closely with the results of the present study, which demonstrated a marked reduction in recurrence risk in the high Ki67 subgroup after chemotherapy (17). Moreover, the distinctive value of CDK4/6 inhibitors in the management of HR+/HER2− breast cancer is intrinsically linked to their ability to suppress tumor proliferation (18), with Ki67 acting as a pivotal biomarker for quantifying this therapeutic effect. Research indicates that CDK4/6 inhibitors markedly suppress tumor cell division and proliferation by inhibiting the transition from the G1 phase to the S phase in the cell cycle (19). Consequently, for patients with high proliferative activity identified by our model, especially when genetic testing is inaccessible, this stratification offers a rationale for screening candidates for future studies investigating intensified adjuvant regimens. Such strategies may include adding CDK4/6 inhibitors to chemotherapy or utilizing CDK4/6 inhibitors in combination with endocrine therapy (20).

Our findings are consistent with evidence from large international clinical trials. The TAILORx trial indicated that, for patients with an intermediate RS, a Ki67 level ≥20% serves as an independent predictive factor for chemotherapy benefit. In addition, within the low RS cohort, the subgroup with high Ki67 expression exhibited a significantly higher 3-year recurrence rate relative to the low Ki67 subgroup (21). Another investigation reported that Ki67 displays a moderate overall correlation with the RS, with a subset of patients (69.8%) having high Ki67 levels but low RS values (22). The RxPONDER trial demonstrated that the efficacy of adjuvant chemotherapy is not solely determined by the RS (23). Collectively, these findings suggest that Ki67 offers risk stratification data independent of genetic testing, representing a crucial biological feature for identifying individuals who are likely to benefit from chemotherapy. By focusing on a node-negative, intermediate-risk patient cohort, our findings further demonstrated that Ki67 and histological grade function as viable surrogate markers in settings where genetic testing is inaccessible. Their predictive utility is consistent with the biological rationale underpinning these trials. In addition, the MINDACT trial showed that chemotherapy may be safely omitted for patients classified as clinically high-risk but genomically low-risk (24). The significance of our stratification model lies in its refinement of the “clinical high-risk” concept using Ki67 and histological grade. This facilitates the identification of patients whose highly proliferative tumor biology strongly indicates a benefit from chemotherapy, even in the absence of genetic testing, thereby promoting more precise therapeutic decisions in settings with constrained resources (25).

When tumor size was analyzed as a continuous variable in our analysis, it emerged as a significant independent prognostic factor. Each 1 cm increment in tumor diameter was associated with a 94% higher risk of DFS recurrence (HR = 1.936) and a twofold increase in the risk of death (HR = 2.143). The discrepancy in the results stemming from the analytical approach underscores that a key limitation of categorical variables is their potential to mask a genuine biological gradient (26). Conversely, modeling tumor size as a continuous variable more precisely captures the inherent dose–response relationship between increasing tumor diameter and escalating risk (27). Notably, even when analyzed as a continuous variable, age did not demonstrate a significant association with the outcomes. This result highlights the pressing need to incorporate analyses using continuous variables into current risk assessment frameworks, which are predominantly based on categorical approaches. Furthermore, our study found that HER2 status was not a significant determinant of patient prognosis. This observation implies that variations in HER2 status might not hold independent prognostic value in this particular cohort of patients with intermediate-risk luminal breast cancer (28).

The risk stratification model developed in this study was based on a single-center cohort, which introduces potential biases. Despite demonstrating robustness through internal bootstrap validation, the model lacks external validation in an independent Chinese population. Therefore, its use should be restricted to research settings at this stage. Standardization of Ki67 assessment remains a persistent challenge worldwide. We acknowledge that inter-observer variability exists in the interpretation of histological grade and Ki67, which can challenge the accuracy and reproducibility of these evaluations. To minimize this variability, we implemented a protocol in which three pathologists conducted independent evaluations, followed by a consensus review. Future research that incorporates digital pathology, whole-slide imaging, and AI-assisted grading tools holds promise for significantly improving the objectivity of these assessments (29). Using OS as a secondary endpoint, rather than breast cancer-specific survival (BCSS), might limit the robustness of our findings. However, in accordance with the STROBE guidelines, OS is still considered a strong endpoint. The fact that all OS events in our cohort occurred after DFS events further substantiates the appropriateness of utilizing OS as an endpoint in this context. However, the low number of OS events resulted in limited statistical power for these analyses. This insufficiency caused substantial variability in the HR estimates and generated abnormally wide confidence intervals in the multivariate analysis for OS, consequently diminishing the statistical robustness and precision of the findings. Consequently, it is imperative to refrain from drawing definitive conclusions from the current OS data. In contrast, the analysis of DFS was based on a comparatively larger number of events, yielding results that are more stable and reliable. Therefore, the primary clinical implication of this study is that the predictive role of these pathological markers for recurrence risk translates into a practical decision-support tool, guiding clinical strategies aimed at improving DFS. In addition, given the relatively favorable prognosis of early-stage luminal breast cancer, extended follow-up periods and larger cohort sizes are required to achieve a sufficient number of survival events and thereby identify potential differences in OS. Of note, certain studies indicate that breast cancers with low ER and PR expression share biological characteristics with classic triple-negative breast cancer (30). To minimize the influence of this subgroup, the present study specifically included only luminal-type patients with ER and/or PR expression levels ≥10%.

In settings where access to genetic testing is limited, the proposed model serves as an effective primary triage strategy. Since Ki67 and histological grade are part of standard pathological assessment, their incorporation into the model is highly cost-effective, imposing minimal additional burden on the healthcare system (25). For patients categorized as high-risk by the model, every effort should be made to recommend genetic testing for further stratification. However, when genetic testing remains inaccessible, strong consideration should be given to administering adjuvant chemotherapy. This approach ensures the allocation of scarce precision medicine resources to patients most likely to benefit, thereby providing a feasible and practical pathway for achieving universally accessible, risk-stratified care.

## 5 Conclusion

Our findings confirm that high histological grade (Grade 2–3) and a Ki67 index ≥20% serve as reliable surrogate indicators of chemotherapy benefit in intermediate-risk, luminal breast cancer patients lacking access to genomic assays. These evidence-based criteria provide a clinically implementable decision-making framework for resource-limited settings, contingent upon the establishment of standardized quality assurance protocols for pathological evaluation. Prospective validation studies are warranted to confirm the predictive accuracy of this model. Future research should also explore combinatorial approaches that integrate next-generation biomarkers, with the ultimate goal of accelerating the democratization of precision oncology.

## Data Availability

The data analyzed in this study is subject to the following licenses/restrictions: the datasets used and/or analysed during the current study are available from the corresponding author on reasonable request. Requests to access these datasets should be directed to YS, 15089231312@163.com.
