# Supplementary material for: A pathology-based surrogate model for chemotherapy decision-making in intermediate-risk luminal breast cancer: validation of histologic grade and Ki67 in a Chinese population
Source: Front Med (Lausanne). 2026 Feb 5;13:1727768. doi: 10.3389/fmed.2026.1727768 (PMC12917894; doi:10.3389/fmed.2026.1727768)
Supplement: Supplementary file 1 [file Supplementary_file_1.docx]

**Figure.1 Sequential Steps in Propensity Score Matching Analysis**


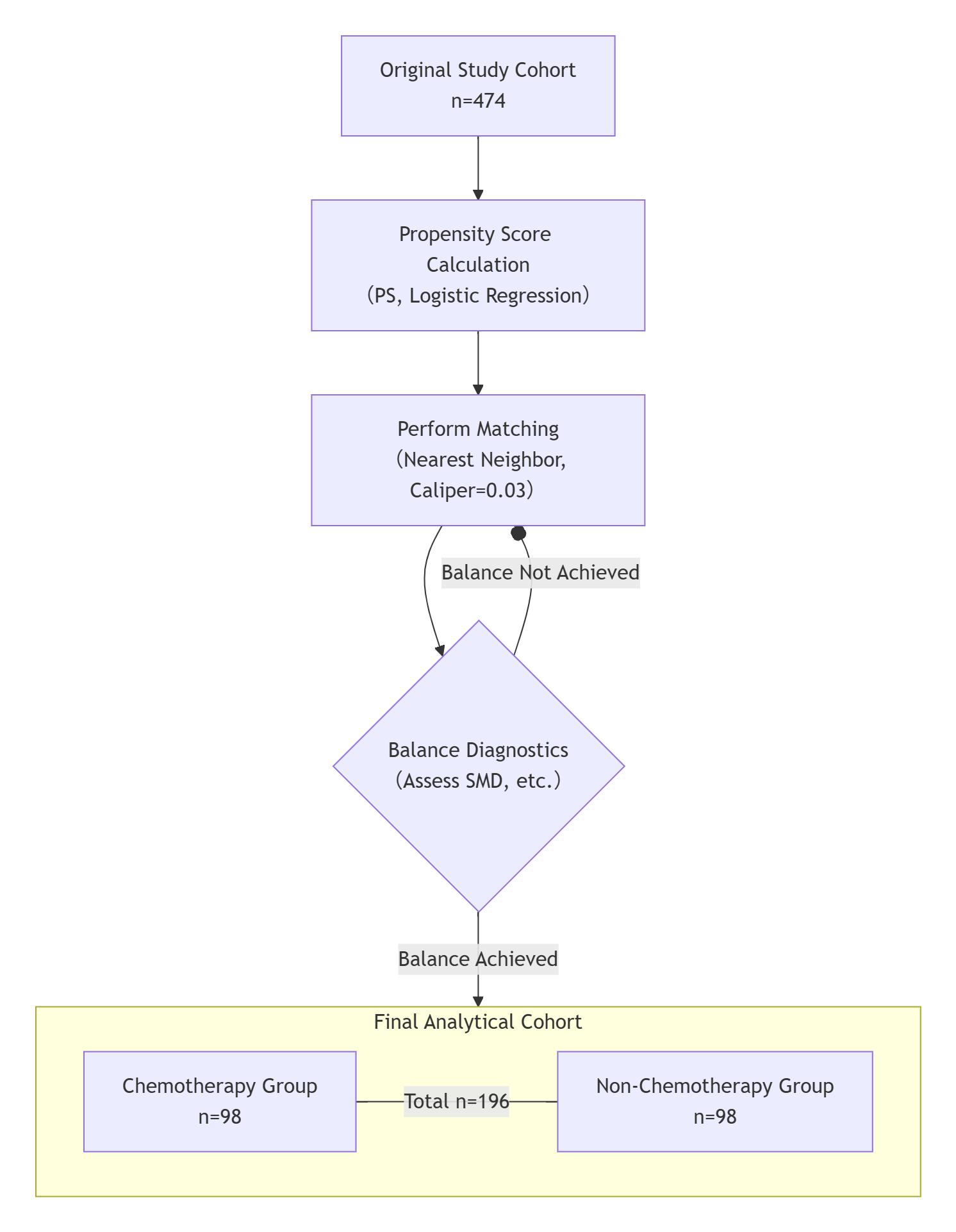


**Table.1 Propensity score matching SMD**

| **Characteristic** | **Propensity score matching**  **Pre-Match Post-Match**  **SMD SMD** | |
| --- | --- | --- |
| Age(years) | 0.220 | 0.104 |
| Menstrual status | 0.275 | 0.206 |
| Breast surgery | 0.066 | 0.043 |
| T Stage | 0.836 | 0.087 |
| Histologic grade | 0.644 | 0.103 |
| PR | 0.334 | 0.088 |
| HER2 Status | 0.036 | 0.190 |
| Ki67 | 0.789 | 0.102 |
| Neural invasion | 0.134 | 0.287 |
| Vascular invasion | 0.660 | 0.024 |
| Endocrine therapy | 0.747 | 0.062 |

**Figure.2**


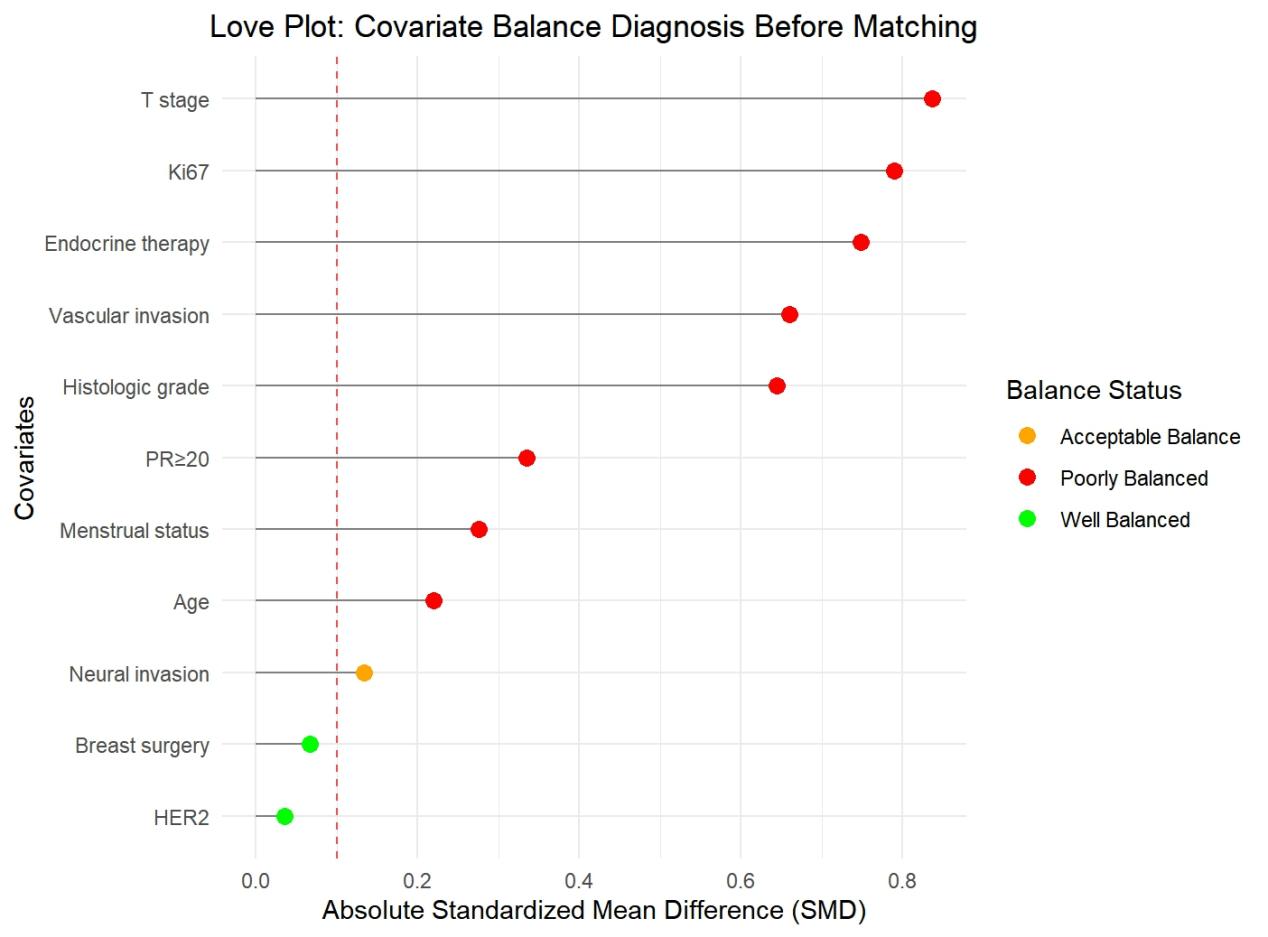


**Figure.3**

**
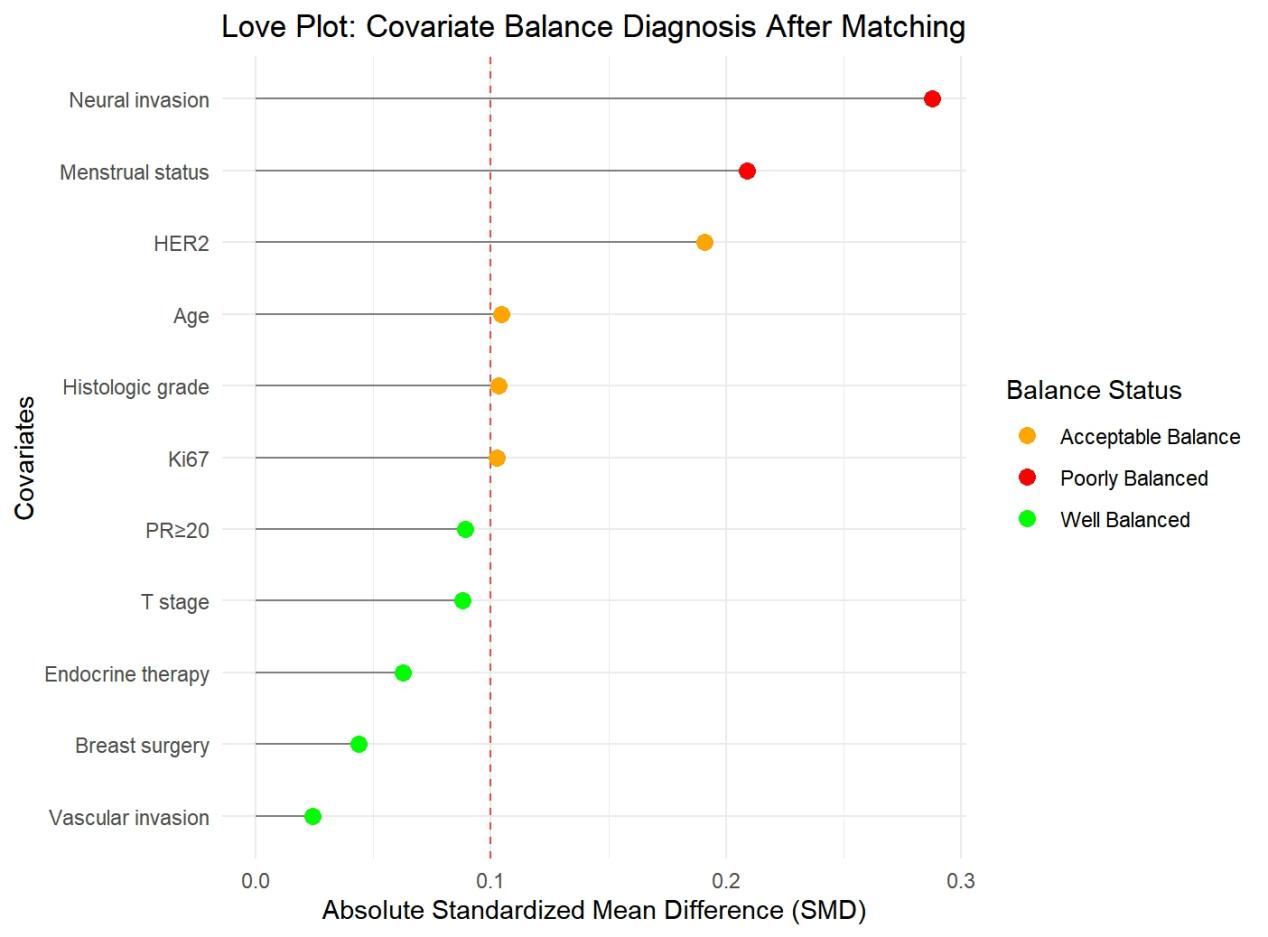
**
