# Supplementary material for: A pathology-based surrogate model for chemotherapy decision-making in intermediate-risk luminal breast cancer: validation of histologic grade and Ki67 in a Chinese population
Source: Front Med (Lausanne). 2026 Feb 5;13:1727768. doi: 10.3389/fmed.2026.1727768 (PMC12917894; doi:10.3389/fmed.2026.1727768)
Supplement: Supplementary file 2 [file Supplementary_file_2.docx]

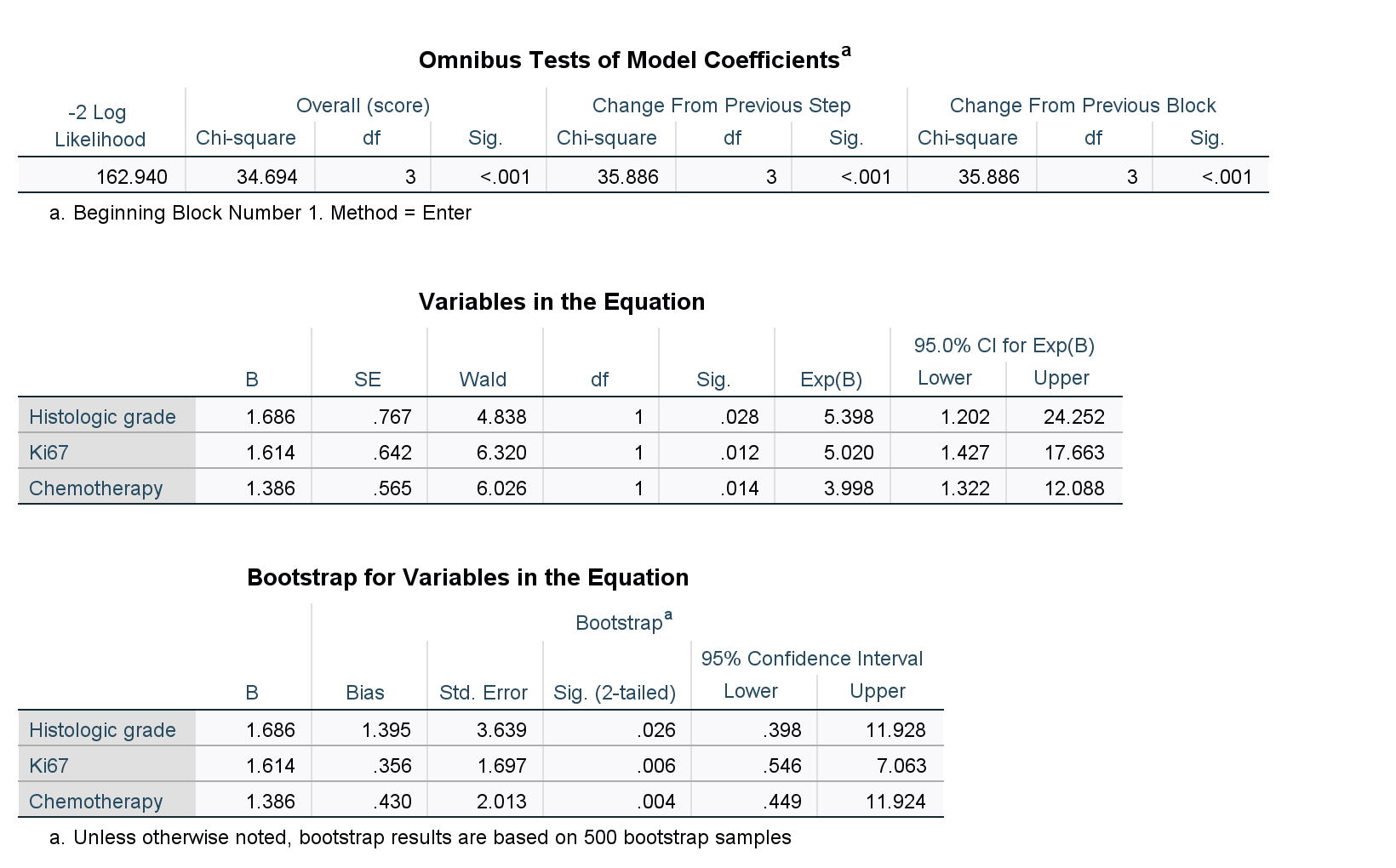
(A)


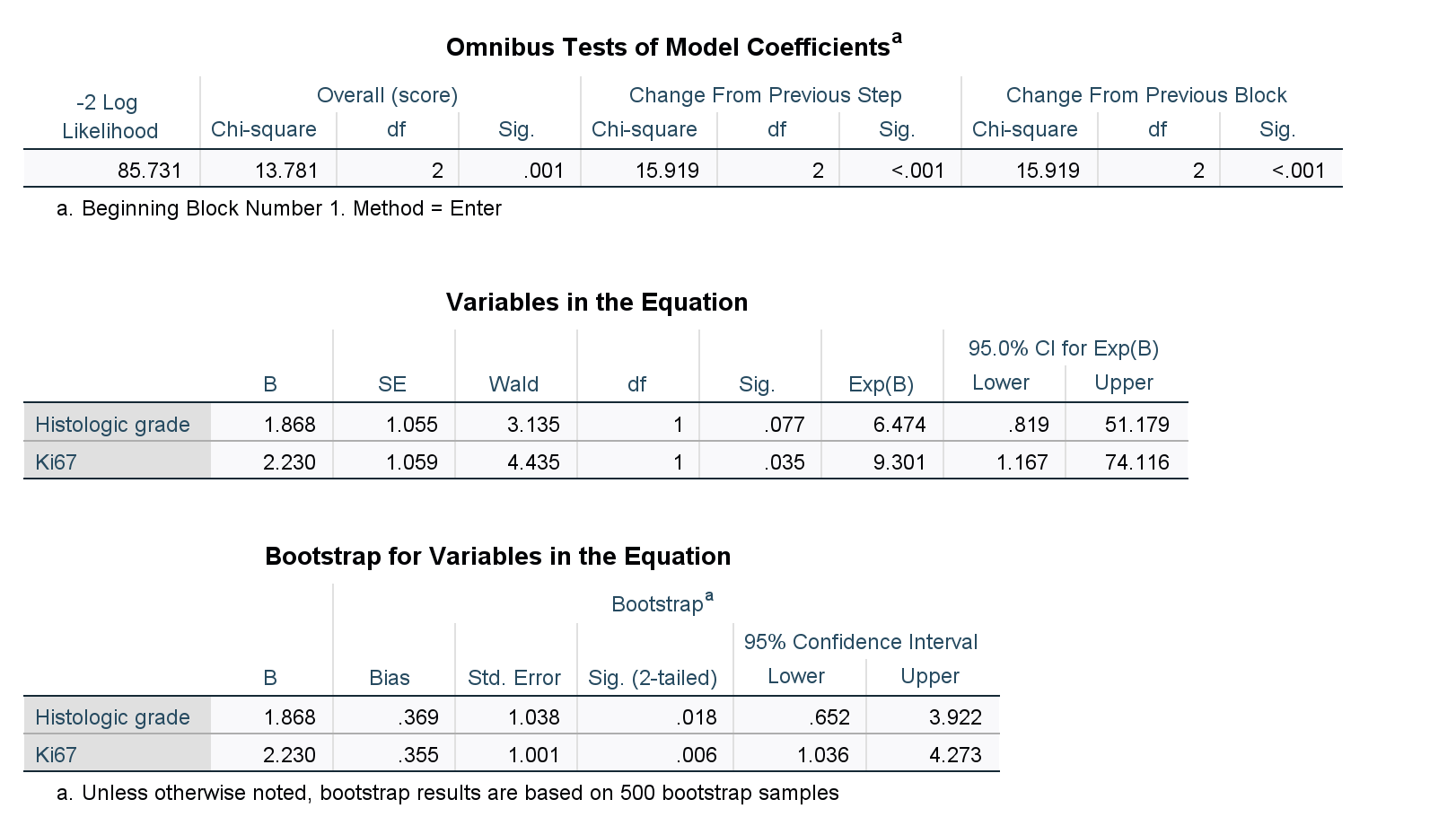


(B)

A: DFS Multivariate Cox Analysis: 500 Bootstrap Sampling Tests

B: OS Multivariate Cox Analysis: 500 Bootstrap Sampling Tests
