# Supplementary material for: A pathology-based surrogate model for chemotherapy decision-making in intermediate-risk luminal breast cancer: validation of histologic grade and Ki67 in a Chinese population
Source: Front Med (Lausanne). 2026 Feb 5;13:1727768. doi: 10.3389/fmed.2026.1727768 (PMC12917894; doi:10.3389/fmed.2026.1727768)
Supplement: Supplementary file 3 [file Supplementary_file_3.docx]

| **Factors** | **Disease-free Survival** | | | |
| --- | --- | --- | --- | --- |
|  | **Univariate analysis** | | **Multivariate analysis** | |
|  | **HR (95% CI)** | **p-value** | **HR (95% CI)** | **p-value** |
| Age(years) | 0.982(0.930,1.036) | 0.501 | ND | ND |
| T size（cm） | 1.837(1.248,2.705) | 0.002 | 1.936(1.194,3.137) | 0.007 |
| Menstrual status |  |  |  |  |
| Premenopausal | 1(Reference) |  | ND | ND |
| Postmenopausal | 0.661(0.280,1.560) | 0.345 | ND | ND |
| Breast surgery |  |  |  |  |
| BCS | 1(Reference) |  | ND | ND |
| Mastectomy | 0.446(0.189,1.051) | 0.065 | ND | ND |
| Histologic grade |  |  |  |  |
| 1 | 1(Reference) |  | 1(Reference) |  |
| 2-3 | 10.567(2.460,45.384) | 0.002 | 3.654(1.202,24.252) | 0.105 |
| PR |  |  |  |  |
| ≥20 | 1(Reference) |  | ND | ND |
| ＜20 | 0.812(0.189,3.488) | 0.779 | ND | ND |
| HER2 Status |  |  |  |  |
| Low | 1(Reference) |  | ND | ND |
| Zero | 1.387(0.572,3.364) | 0.469 | ND | ND |
| Ki67 |  |  |  |  |
| ＜20% | 1(Reference) |  | 1(Reference) |  |
| ≥20 | 8.058(2.364,27.474) | ＜0.001 | 5.337(1.478,19.272) | 0.011 |
| Neural invasion |  |  |  |  |
| Yes | 1(Reference) |  | ND | ND |
| No | 0.585(0.227,1.509) | 0.267 | ND | ND |
| Vascular invasion |  |  |  |  |
| Yes | 1(Reference) |  | ND | ND |
| No | 0.633(0.262,1.530) | 0.310 | ND | ND |
| Endocrine therapy |  |  |  |  |
| TAM | 1(Reference) |  | ND | ND |
| AI | 1.506(0.639,3.550) | 0.349 | ND | ND |
| Chemotherapy |  |  |  |  |
| Yes | 1(Reference) |  | 1(Reference) |  |
| No | 5.816(1.952,17.329) | 0.002 | 4.645(1.471,14.668) | 0.009 |

Note：HER2, human epidermal growth factor receptor 2; PR, progesterone receptor; BCS Breast

conserving surgery; TAM：Tamoxifen；AI：Aromatase inhibitors
