# Supplementary material for: A pathology-based surrogate model for chemotherapy decision-making in intermediate-risk luminal breast cancer: validation of histologic grade and Ki67 in a Chinese population
Source: Front Med (Lausanne). 2026 Feb 5;13:1727768. doi: 10.3389/fmed.2026.1727768 (PMC12917894; doi:10.3389/fmed.2026.1727768)
Supplement: Supplementary file 4 [file Supplementary_file_4.docx]

Cox proportional hazard model of overall survival (OS) in PSM group

| **Factors** | **Overall Survival** | | | |
| --- | --- | --- | --- | --- |
|  | **Univariate analysis** | | **Multivariate analysis** | |
|  | **HR (95% CI)** | **p-value** | **HR (95% CI)** | **p-value** |
| Age(years) | 0.975(0.905,1.051) | 0.508 | ND | ND |
| T size（cm） | 2.161(1.288,3.628) | 0.004 | 2.143(1.204,3.814) | 0.010 |
| Menstrual status |  |  |  |  |
| Premenopausal | 1(Reference) |  | ND | ND |
| Postmenopausal | 1.456(0.443,4.782) | 0.536 | ND | ND |
| Breast surgery |  |  |  |  |
| BCS | 1(Reference) |  | ND | ND |
| Mastectomy | 0.404(0.123,1.323) | 0.134 | ND | ND |
| Histologic grade |  |  |  |  |
| 1 | 1(Reference) |  | 1(Reference) |  |
| 2-3 | 9.800(1.254,76.589) | 0.030 | 5.170(0.653,40.928) | 0.120 |
| PR |  |  |  |  |
| ≥20 | 1(Reference) |  | ND | ND |
| ＜20 | 0.041(0.000,152,552) | 0.447 | ND | ND |
| HER2 Status |  |  |  |  |
| Low | 1(Reference) |  | ND | ND |
| Zero | 1.172(0.339,4.053) | 0.802 | ND | ND |
| Ki67 |  |  |  |  |
| ＜20% | 1(Reference) |  | 1(Reference) |  |
| ≥20 | 12.819(1.638,100.356) | 0.015 | 10.538(1.258,88.251) | 0.030 |
| Neural invasion |  |  |  |  |
| Yes | 1(Reference) |  | ND | ND |
| No | 0.948(0.205,4.390) | 0.945 | ND | ND |
| Vascular invasion |  |  |  |  |
| Yes | 1(Reference) |  | ND | ND |
| No | 0.718(0.209,2.461) | 0.598 | ND | ND |
| Endocrine therapy |  |  |  |  |
| TAM | 1(Reference) |  | ND | ND |
| AI | 1.434(0.437,4.702) | 0.552 | ND | ND |
| Chemotherapy |  |  |  |  |
| Yes | 1(Reference) |  | ND | ND |
| No | 3.208(0.850,12.105) | 0.085 | ND | ND |

Note：HER2, human epidermal growth factor receptor 2; PR, progesterone receptor; BCS Breast

conserving surgery; TAM：Tamoxifen；AI：Aromatase inhibitors
