# Supplementary figures and images for: A pathology-based surrogate model for chemotherapy decision-making in intermediate-risk luminal breast cancer: validation of histologic grade and Ki67 in a Chinese population
Source: Front Med (Lausanne). 2026 Feb 5;13:1727768. doi: 10.3389/fmed.2026.1727768 (PMC12917894; doi:10.3389/fmed.2026.1727768)

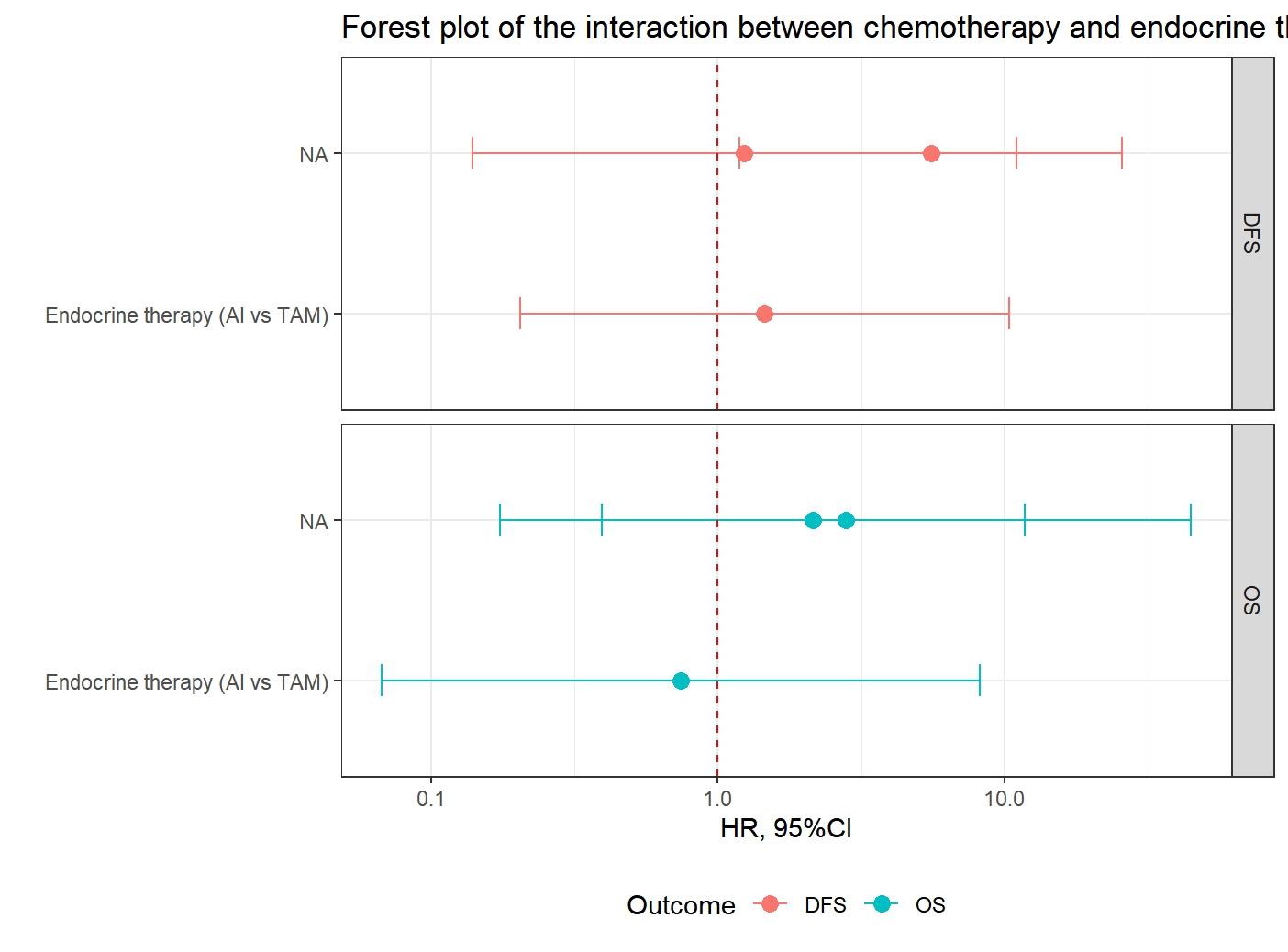

Supplement: Supplementary file 5 [file Supplementary_file_5.jpeg]
